# The Prevalence of Gastric Atrophy in Western and Northern European Populations: A Systematic Review and Meta-Analysis

**DOI:** 10.1007/s12029-025-01291-z

**Published:** 2025-08-19

**Authors:** Eoghan Burke, Patricia Harkins, Mayilone Arumugasamy

**Affiliations:** 1 The Royal College of Surgeons in Ireland, Dublin, Ireland; 2 St James’s Hospital, Dublin, Ireland; 3 Mater Misericordiae University Hospital, Dublin, Ireland; 4 Beaumont Hospital, Dublin, Ireland

**Keywords:** Gastric atrophy, Gastric cancer, Screening, Epidemiology

## Abstract

**Background:**

Gastric atrophy (GA) is a pre-neoplastic condition leading to gastric cancer (GC). Early GA detection is critical for guiding surveillance and preventing advanced GC. Histology is the current gold standard for GA diagnosis, but is considered not cost-effective for routine screening in Western populations. Serological methods offer a potentially affordable alternative. Understanding GA prevalence, symptom impact, and optimal detection strategies in low-risk Western populations is essential before integrating GA screening into GC prevention programs.

**Methods:**

This systematic review and meta-analysis assessed GA prevalence in Northern and Western European populations. Key outcomes included GA prevalence (any topographical distribution in the stomach and corpus-specific), effects of symptomatology on prevalence, and differences between serological and histological prevalence.

**Results:**

Twenty-two cross-sectional studies (*n* = 62,520) were included; 13 used histology and 9 used serology. Overall GA prevalence of any topographical distribution was 13% (95% confidence interval (CI) 7–18%). Histology-based studies reported 21% (95% CI 11–30%) versus 5% (95% CI 3–7%) by serology.

Corpus-involving GA had a pooled prevalence of 6% (95% CI 4–9%), with histology detecting higher rates (10–15%) than serology (4–5%).

In symptomatic populations, GA prevalence rose to 47%, compared to 6–10% in asymptomatic groups. Corpus GA reached 20% in symptomatic patients versus 6–8% in asymptomatic ones.

**Conclusion:**

GA, especially corpus-involving GA, is more prevalent in Western and Northern European populations than previously thought. These findings suggest that screening for GA in these populations may be a viable route to increasing early GC detection rates and improving outcomes.

## Introduction

Gastric atrophy (GA) is a pre-cancerous condition affecting the stomach [1]. It forms part of the inflammation–atrophy–metaplasia–dysplasia–carcinoma pathway known as the Correa cascade [2]. It is characterised by the loss of the normal glandular architecture of the stomach [3] and is most commonly caused by *Helicobacter pylori*-induced chronic gastritis [4].

Identifying patients with GA is a clinical priority both to guide surveillance and to intervene in preventing advanced gastric cancer (GC) [5]. Traditionally, GA was associated solely with the development of intestinal type gastric cancer (ITGC); however, recent evidence suggests it may also play a role in the pathogenesis of diffuse type gastric cancer (DTGC) [6]. Eradication of *Helicobacter pylori* in patients with GA has been shown to reverse GA and reduce the risk of progression to GC [7]. However, the eradication of *Helicobacter pylori* in patients with established intestinal metaplasia (IM) has not shown the same benefit, highlighting the importance of early detection of GA [8]. This has prompted a search for non-invasive techniques to screen populations to identify patients with GA and, thus, at risk of GC.

Pepsinogen I, the pepsinogen I:II ratio, gastrin-17, and *Helicobacter pylori* antigen show high sensitivity and specificity for detecting GA in GC high-risk populations, particularly in Asia [9]. However, data on GA prevalence in lower-risk GC populations, such as those in Western and Northern Europe, are limited, thus impacting the design of diagnostic accuracy studies to assess serological-based GA diagnosis in these populations [10]. Existing studies suggest a prevalence of 10–18.5% [11], but more precise estimates are needed to guide cost-effective GC screening strategies. To address this gap, we conducted the first systematic review and meta-analysis to assess GA prevalence in these low-risk regions and inform future research and clinical guidelines.

## Study Aims

This systematic review aims to synthesize for the first time all of the available evidence assessing the prevalence of GA in GC low-risk Western populations, specifically focused on Northern and Western European populations.

Outcomes of interest included the prevalence rate of GA regardless of topographical distribution in the stomach, the prevalence rate of GA affecting the corpus of the stomach, the effects of symptomatology on prevalence rates, and the differences in prevalence rates reported by histological assessment and serological assessment.

## Methods

### Study Design

This is a systematic review of studies assessing the prevalence of GA in Northern and Western European populations. The reporting of this systematic review is in accordance with the Preferred Reporting Items for Systematic Reviews and Meta-Analyses (PRISMA) statement [12]. This systematic review was registered prospectively with the PROSPERO database, registration number: CRD42024570311.

### Inclusion and Exclusion Criteria

All studies addressing the prevalence rates of GA in Northern and Western European populations were considered for inclusion. Only studies available in the English language were assessed. Studies reporting the prevalence rates based on histological and serological diagnosis were considered. Bibliographic databases were searched from inception until January 2025.

This study defined Northern and Western European countries as those classified by the Global Cancer Observatory (GLOBOCAN), which is part of the International Agency for Research on Cancer, which is part of the World Health Organization (WHO). By GLOBOCAN definitions, Northern and Western European countries include Finland, Sweden, Norway, Denmark, Greenland, Iceland, Germany, Austria, Belgium, Luxembourg, Liechtenstein, Switzerland, the Netherlands, France, the UK, and Ireland [13, 14]. The Baltic states were excluded due to the recognition that the Baltic states are more closely aligned with those of Eastern European countries socioeconomically and so would share similar etiological factors and thus incidence and prevalence rates of GC [15, 16].

Studies addressing the prevalence of GA in GC high-risk countries, those available only as abstracts where sufficient data could not be extracted, and those involving children were excluded. Studies describing the prevalence of GA in the setting of established GC were excluded as they would artificially raise the prevalence and are not representative of a general population-based prevalence of GA.

If a study included populations from both a suitable low-risk country and an unsuitable high-risk country, the data pertaining to the low-risk country were extracted for inclusion where possible.

### Search Strategy

A search strategy was developed in coordination with a medical librarian. The search string utilized keywords and MeSH terms for “prevalence” and “gastric atrophy” or “atrophic gastritis.” This strategy was chosen to keep the search sufficiently broad to capture all relevant studies. The search string was applied to the following bibliographic databases: PubMed, EMBASE, and Web of Science. All databases were searched from inception with no language restriction until January 2025.

### Study Selection

The studies identified from the search strategy were uploaded to a systematic review management software package [17]. After removing duplicates, two authors reviewed all the titles and abstracts independently. Those meeting the aforementioned inclusion criteria were brought forward for full-text review. Any conflicts concerning a study’s suitability were resolved by consensus. Following a full-text review, all suitable studies were brought forward for qualitative review. Hand-searching of references in the included studies was completed to identify any missed studies. Similarly, a citation search using Google Scholar was used to ensure no studies were omitted from the qualitative analysis.

### Data Extraction

Two authors independently extracted the required data from the eligible studies using a predetermined data extraction form. Any conflicts in the extracted data were resolved by consensus. The data extracted included the authors, year of publication, study design, number of patients, patient demographics, and the prevalence of GA.

### Risk of Bias Assessment

Two of the authors independently assessed the risk of bias in each of the included studies using the AXIS tool for assessing cross-sectional studies [18, 19].

### Summary Measures and Synthesis of Results

This systematic review’s outcome of interest was the prevalence rate of GA in Western and Northern European populations. Prevalence rates were converted to proportions to allow for meta-analysis between suitable studies. The effect size was expressed as a proportional prevalence rate with the standard error (SE) calculated using the equation SE = √p(1-p)/n where p is the sample proportion (prevalence proportion) and n is the sample size. Meta-analysis was performed using the Meta-Essentials software package [20]. *P* value < 0.05 was considered statistically significant.

## Results

### Study Selection

**Fig. 1:**
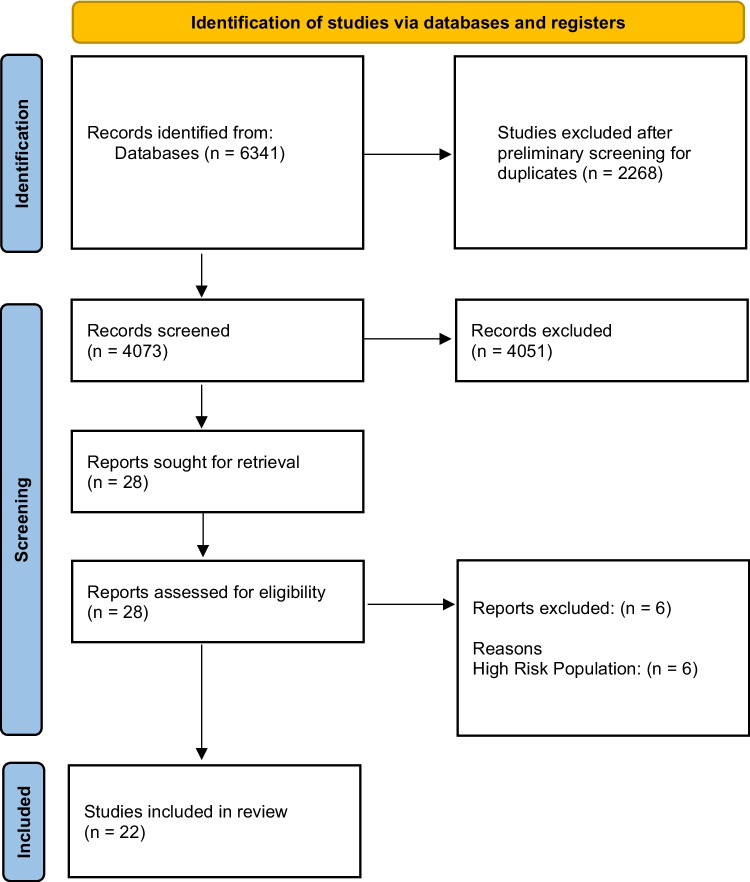
PRISMA flow chart depicting the study selection process

After applying the search string to the respective bibliographic databases, 6341 articles were identified. Following the identification and removal of duplicate studies/papers, 4073 articles remained. Following a screening of the titles and abstracts of these articles, 4051 were excluded as they did not meet the aforementioned inclusion criteria. Twenty-eight full articles were sought for review. Following a full review of these articles, 6 were excluded as they concerned high-risk populations and not the populations of interest for this study. This resulted in 22 unique studies being suitable for inclusion in qualitative and quantitative review. The PRISMA flow chart is depicted in Fig. 1.

### Study Characteristics

Tables 1 and 2 outline the characteristics of the included studies. The final 22 included studies were published between 1985 and 2021 [21–42]. Finland was the country of origin of most of the studies, numbering 8 in total [21–24, 26, 28, 36, 39], followed by Sweden (*n* = 4) [32, 33, 38, 40], Germany (*n* = 3) [25, 41, 42], the Netherlands (*n* = 3) [29, 35, 37], the UK (*n* = 2) [27, 30], and France (*n* = 2) [31, 34]. All of the included studies were cross-sectional in nature, with the majority being prospective [23–26, 28–32, 34, 35, 37–42] and only five being retrospective [21, 22, 27, 33, 36]. The 22 included studies involved a total of 62,520 patients, with 44,571 males and 12,090 females, with a mean age across the studies of 58. Notably, three studies did not give a breakdown of the male-to-female ratio [33, 39, 42], and four did not give a breakdown of age [24, 26, 29, 33].

**Table 1:** Characteristics of included studies, including the year the study was conducted, country of origin, study design, number of patients, histological assessment, serological assessment, histology reporting system used, serology markers used, and cutoff value of pepsinogen 1 used. *NR* not reported. *N/A* not applicable

Study ID	Year	Country of origin	Study design	Retrospective or prospective	Number of patients	Histological assessment	Serological assessment	Histology reporting system used	Biomarkers assessed	Pepsinogen 1 cutoff value
Sipponen et al. [21]	2021	Finland	Cross-sectional	Retrospective	2298	Yes	No	Sydney	N/A	N/A
Paloheimo et al. [22]	2021	Finland	Cross-sectional	Retrospective	500	No	Yes	N/A	pepsinogen1, pepsinogen 2, pepsinogen 1:2 ratio, gastrin 17 and Igg H.Pylori	< 30 µg/L
Oksanen et al. [23]	2000	Finland	Cross-sectional	Prospective	207	Yes	Yes	Sydney	Igg H.P, Gastrin, Pepsinogen 1, parietal antibodies, Cag A	< 28 µg/L
Härkönen et al. [24]	2003	Finland	Cross-sectional	Prospective	12,252	No	Yes	N/A	Pepsinogen 1	< 25 µg/L
Weck et al. [25]	2009	Germany	Cross-sectional	Prospective	9444	No	Yes	N/A	pepsinogen 1, pepsinogen 1:2 ratio	< 70 µg/L
Siurala et al. [26]	1985	Finland	Cross-sectional	Prospective	371	Yes	No	other	N/A	N/A
Knight et al. [27]	1996	UK	Cross-sectional	Retrospective	54	Yes	Yes	other	pepsinogen 1	< 150 µg
Telaranta-Keerie et al. [28]	2010	Finland	Cross-sectional	Prospective	4256	No	yes	N/A	pepsinogen1, pepsinogen2, pepsinogen 1:2 ratio, Igg H.Pylori	< 30 µg/L
Schlemper et al. [29]	1995	Netherlands	Cross-sectional	Prospective	498	No	Yes	N/A	pepsinogen1, pepsinogen 2, pepsinogen 1:2 ratio, gastrin and Igg H.Pylori	< 19 µg/L
Sitas et al. [30]	1993	UK	Cross-sectional	Prospective	87	Yes	No	Whiteheads classification	N/A	N/A
Potet et al. [31]	1993	France	Cross-sectional	Prospective	742	Yes	No	Whiteheads classification	N/A	N/A
Storskrubb et al. [32]	2008	Sweden	Cross-sectional	Prospective	976	Yes	Yes	Sydney	pepsinogen1, pepsinogen 2, pepsinogen 1:2 ratio, gastrin and Igg H.Pylori	< 25 µg/L
Song et al. [33]	2015	Sweden	Cross-sectional	Retrospective	5284	No	Yes	N/A	pepsinogen 1	< 28 µg/L and < 45 µg/L
Chapelle et al. [34]	2020	France	Cross-sectional	Prospective	344	Yes	Yes	Sydney	pepsinogen1, pepsinogen 2, pepsinogen 1:2 ratio, gastrin 17 and Igg H.Pylori	< 30 µg/L
Korstanjea et al. [35]	2006	Netherlands	Cross-sectional	Prospective	997	No	Yes	N/A	pepsinogen1, pepsinogen 2, pepsinogen 1:2 ratio, gastrin and Igg H.Pylori	< 17 µg/L
Varis et al. [36]	2000	Finland	Cross-sectional	Retrospective	22,436	Yes	Yes	Sydney	Pepsinogen 1	< 25 µg/L
den Hoed et al. [37]	2010	Netherlands	Cross-sectional	Prospective	383	Yes	No	Sydney	N/A	N/A
Zuzek et al. [38]	2011	Sweden	Cross-sectional	Prospective	368	Yes	No	NR	N/A	N/A
Aine et al. [39]	2016	Finland	Cross-sectional	Prospective	106	No	Yes	N/A	pepsinogen1, pepsinogen 2, pepsinogen 1:2 ratio, gastrin 17 and Igg H.Pylori	NR
Lewerin et al. [40]	2008	Sweden	Cross-sectional	Prospective	190	No	Yes	N/A	pepsinogen1, pepsinogen 2, pepsinogen 1:2 ratio, gastrin 17 and Igg H.Pylori	< 30 µg/L
Venerito et al. [41]	2010	Germany	Cross-sectional	Prospective	258	Yes	Yes	Sydney	pepsinogen1, pepsinogen 2, pepsinogen 1:2 ratio, gastrin 17 and Igg H.Pylori	NR
Selgrad et al. [42]	2015	Germany	Cross-sectional	Prospective	469	Yes	Yes	Sydney	pepsinogen1, pepsinogen 2, pepsinogen 1:2 ratio, gastrin 17 and Igg H.Pylori	NR

**Table 2:** Characteristics of included studies including demographic details, prevalence of gastric atrophy (GA) of any topographical distribution and its corresponding prevalence proportion and prevalence of Corpus GA and its corresponding prevalence proportion.*NR* not reported

Study ID	Total patients	Males	Females	Mean age	Patients with gastric atrophy (any topographic distribution)	Proportional prevalence rate of gastric atrophy of any topographical distribution	Patients with gastric atrophy affecting the corpus	Proportional prevalence rate of corpus GA
Sipponen et al. [21]	2298	1130	1168	49	698	0.303742385	NR	
Paloheimo et al. [22]	500	147	353	59.9	14	0.028	14	0.028
Oksanen et al. [23]	207	85	122	55	52	0.251207729	16	0.077294686
Härkönen et al. [24]	12,252	12,252	0	NR	635	0.051828273	635	0.051828273
Weck et al. [25]	9444	4244	5200	62	535	0.056649725	NR	
Siurala et al. [26]	371	190	181	NR	137	0.369272237	60	0.161725067
Knight et al. [27]	54	54	0	43	9	0.166666667	9	0.166666667
Telaranta-Keerie et al. [28]	4256	1533	2723	56	150	0.035244361	150	0.035244361
Schlemper et al. [29]	498	425	73	NR	8	0.016064257	NR	
Sitas et al. [30]	87	52	35	47.5	48	0.551724138	22	0.252873563
Potet et al. [31]	742	386	356	53	206	0.277628032	17	0.022911051
Storskrubb et al. [32]	976	473	503	54	62	0.06352459	62	0.06352459
Song et al. [33]	5284	NR	NR	nr	305	0.057721423	305	0.057721423
Chapelle et al. [34]	344	156	188	58.8	148	0.430232558	77	0.223837209
Korstanjea et al. [35]	997	455	542	52	34	0.034102307	34	0.034102307
Varis et al. [36]	22,436	22436	0	57	1044	0.046532359	1044	0.046532359
den Hoed et al. [37]	383	191	192	53	30	0.078328982	NR	
Zuzek et al. [38]	368	176	192	54	11	0.029891304	NR	
Aine et al. [39]	106	NR	NR	82	13	0.122641509	NR	
Lewerin et al. [40]	190	76	114	76	26	0.136842105	8	0.042105263
Venerito et al. [41]	258	110	148	58	25	0.096899225	14	0.054263566
Selgrad et al. [42]	469	NR	NR	67	40	0.085287846	40	0.085287846

GA was diagnosed using the gold standard of histological assessment of biopsies taken by endoscopy in 13 of the included studies [21, 23, 26, 27, 30–32, 34, 36–38, 41, 42] and via serological assessment alone in 9 studies [22, 24, 25, 28, 29, 33, 35, 39, 40]. There was heterogeneity among the studies in terms of which histological reporting system was used to report GA and which serological markers and cutoff values thereof were used in the serological-based studies (Table 1). A number of studies used a combination of histological and serological assessments; however, where this occurred, the histological result was taken as the true value of GA prevalence.

The topographical distribution of GA was not consistently reported across all studies. Sixteen studies reported the prevalence of Corpus GA, which is considered the higher risk form of GA, as distinct from distal or antral predominant GA [22–24, 26–28, 30–36] [40] [41] [42]. Table 2 outlines the prevalence of GA, regardless of topographical distribution, and of Corpus GA where reported. The proportional prevalence rates are also reported for each study.

### Risk of Bias Assessment

Two authors independently appraised each included study for risk of bias using the AXIS tool specifically designed for the critical appraisal of cross-sectional studies [19]. All of the included studies were deemed to be of low risk of bias, with the exception of the five studies, which were performed retrospectively [21, 22, 27, 33, 36]. These five studies were deemed to be of moderate risk of bias.

A funnel plot analysis was conducted for the meta-analysis assessing the prevalence of GA regardless of topographical distribution in the stomach and for the meta-analysis of the prevalence of GA affecting the corpus of the stomach (Appendices 1 and 2). Both funnel plots displayed asymmetry, being skewed to the right, suggesting publication bias. The adjusted combined effect size was closer to zero in both, suggesting the reported pooled effects may be overestimated due to missing negative or null results.

### Prevalence of Gastric Atrophy of any Topographical Distribution

**Fig. 2:**
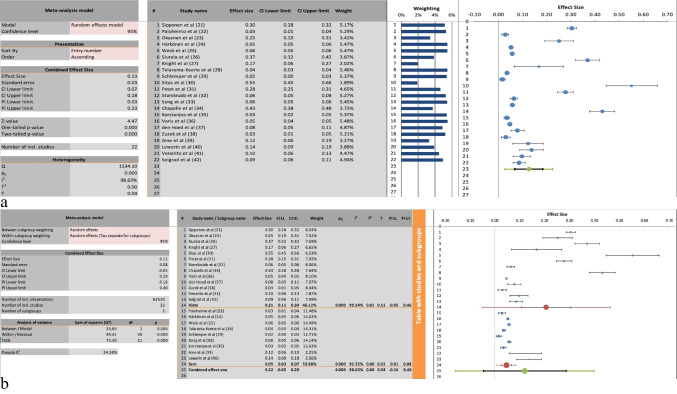
**a** The meta-analysis of the prevalence of gastric atrophy regardless of topographical distribution. **b** A subgroup analysis divided by detection method (histological versus serological)

All of the included studies reported on the prevalence of GA; however, the precise topographical distribution was not always reported. Figure 2a depicts the meta-analysis of the prevalence of GA regardless of topographical distribution in the stomach. The pooled effect size for this meta-analysis was 0.13 with a 95% confidence interval (CI) from 0.07 to 0.18, which was statistically significant, *P*-value < 0.00. Significant heterogeneity was found in the included studies with an I^2^ value of 99%.

Figure 2b displays a subgroup analysis of the prevalence of GA regardless of topographical distribution in the stomach, with subgroups divided depending on the method of analysis, namely histological assessment or serological assessment. The histological assessment subgroup, comprised of 13 studies, had an overall effect size of 0.21 with a 95% CI between 0.11 and 0.3, which was statistically significant, *P*-value < 0.00. This subgroup had significant heterogeneity, with an I^2^ of 99%. The overall effect size for the serological group, which comprised nine studies, was 0.05, with a 95% CI between 0.03 and 0.07, which was statistically significant, *P*-value < 0.00. This subgroup had significant heterogeneity, with an I^2^ of 93%.

### Prevalence of Gastric Atrophy Affecting the Corpus of the Stomach

**Fig. 3:**
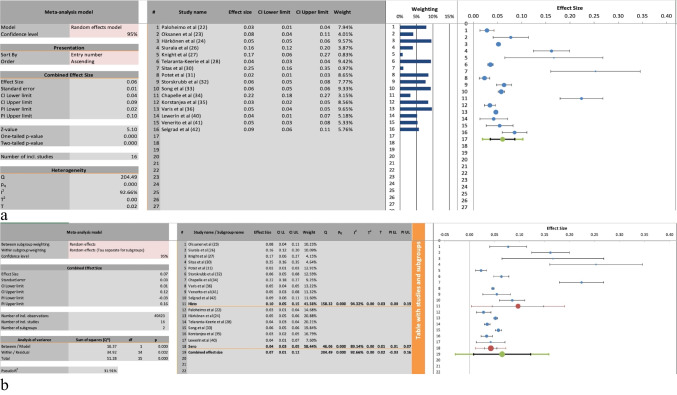
**a** The meta-analysis of the prevalence of gastric atrophy involving the corpus. **b** The subgroup analysis stratified based on detection method (histological versus serological)

Figure 3a depicts the meta-analysis of the prevalence of GA affecting the Corpus; 16 of the included studies reported on this finding [22–24, 26–28, 30–36, 40–42]. The overall effect size was 0.06 with a 95% CI between 0.04 and 0.09, which was statistically significant, *P*-value < 0.00. There was significant heterogeneity between the included studies, with an I^2^ of 93%.

Figure 3b depicts the subgroup analysis of the previously mentioned meta-analysis, with subgroups divided based on the assessment methods, namely histological assessment or serological assessment. Ten of the included studies assessed the prevalence of corpus GA using histological assessment of biopsies; the pooled effect size for this subgroup was 0.1 with a 95% CI between 0.05 and 0.15, which was statistically significant, *P*-value < 0.00. There was significant heterogeneity in this subgroup, I^2^ of 94%.

Serological assessment was used to assess the prevalence of corpus GA in six of the included studies. The pooled effect size for this subgroup was 0.04 with a 95% CI between 0.03 and 0.05, which was statistically significant, *P*-value < 0.00. There was significant heterogeneity between the studies in this subgroup, I^2^ of 89%.

### Prevalence of Gastric Atrophy Stratified by Symptomatology

**Fig. 4:**
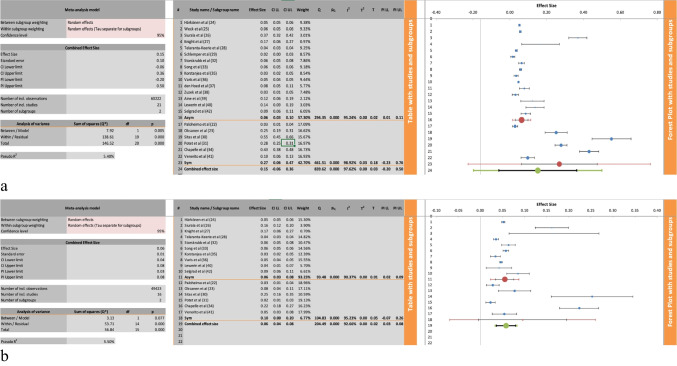
** a** The meta-analysis for the prevalence of gastric atrophy of any topographical distribution with subgroup analysis by symptomatology. **b** The meta-analysis of the prevalence of gastric atrophy involving the corpus with subgroup analysis by symptomatology

Figure 4a depicts the meta-analysis of the prevalence of GA of any topographical distribution with a subclassification stratified by symptomatology. Symptomatic cohorts were defined as patients presenting for testing (either via gastroscopy with biopsy or via serological assessment) to investigate upper gastrointestinal symptoms such as dyspepsia and reflux. Asymptomatic cohorts were taken to be patients tested as part of a screening study, and so by definition were asymptomatic. All of the included studies reported on these variables; however, Sipponen et al. [21] was excluded from this meta-analysis as this study assessed a mixed cohort of both symptomatic and asymptomatic patients, with 41% of the asymptomatic patients being first-degree relatives of GC patients and so being at higher risk of GC and thus higher risk of GC precursor lesions by default. On subgroup analysis, six studies assessed the prevalence of GA regardless of topographical distribution in symptomatic patients [22, 23, 30, 31, 34, 41], yielding an effect size of 0.27 with a 95% CI from 0.06 to 0.47, which was statistically significant with a *P*-value of 0.00. This subgroup had significant heterogeneity with an I^2^ value of 99%.

The prevalence of GA, regardless of topographical distribution, was assessed in asymptomatic cohorts in 15 of the included studies [24–29, 32, 33, 35–40, 42]. The pooled effect size for this subgroup was 0.06 with a 95% CI between 0.03 and 0.1, which was statistically significant with a *P*-value of 0.00. There was significant heterogeneity within this subgroup, I^2^ value of 95%.

Figure 4b depicts the meta-analysis for the prevalence of corpus predominant GA with a subgroup analysis stratified into symptomatic and asymptomatic. Of the included studies, 16 reported on the prevalence of GA affecting the corpus, with a subgrouping by symptoms. The symptomatic subgroup comprised six studies [22, 23, 30, 31, 34, 41] with a pooled effect size of 0.1 with a 95% CI between 0.00 and 0.2, which was statistically significant, *P*-value 0.00. This subgroup had significant heterogeneity, with an I^2^ value of 95%.

The asymptomatic subgroup comprised ten studies with a pooled effect size of 0.06 with a 95% CI between 0.03 and 0.08, which was statistically significant, *P*-value 0.00. There was significant heterogeneity within this subgroup, I^2^ value of 90%.

## Discussion

This systematic review and meta-analysis is the first to comprehensively assess the prevalence of GA in Western and Northern European populations. The study aimed to evaluate four key areas: the overall prevalence of GA regardless of topographical distribution in the stomach; the prevalence of GA involving the gastric corpus, which carries a higher risk of progression to GC; differences in prevalence between symptomatic and asymptomatic individuals; and the impact of detection method, histology versus serology, on reported prevalence rates.

A total of 22 studies were included, encompassing 62,520 patients with a mean age of 58. Some studies did not report complete demographic information [24, 26, 29, 33, 39, 42]. The overall pooled prevalence of GA, regardless of its location in the stomach, was 13%, with a 95% CI of 7% to 18% (Fig. 2a). When broken down by detection method, studies reporting histology reported a prevalence of 21% (CI 11–30%), compared to just 5% (CI 3–7%) in studies using serology (Fig. 2b). This suggests that the true prevalence of GA in Western and Northern European populations may be as high as 30% when using the gold standard of histological diagnosis, significantly higher than previously reported estimates [11]. The subgroup analysis discrepancy between detection methods highlights a potential underestimation of GA prevalence in studies relying solely on serological testing, although the heterogeneity in cutoff values for the serological markers used in the studies warrants caution in interpreting these results.

In terms of corpus-involving GA, which is considered more clinically significant due to its association with increased risk of GC [1], the pooled prevalence across 16 studies was 6% (CI 4–9%) (Fig. 3a). Again, prevalence was notably higher in histological assessments (10–15%) compared to serological assessments (4–5%) (Fig. 3b). These findings reinforce the concern that serological studies may underreport true disease prevalence. Again, a limitation of our study was the heterogeneity in the cutoff values used by the included studies for the serological diagnosis of GA. Again, this warrants caution when interpreting these results.

When comparing symptomatic and asymptomatic cohorts, significant differences emerged. Among symptomatic individuals, the prevalence of GA (regardless of location in the stomach) reached up to 47%, based on data from six studies (Fig. 4a). In contrast, 15 studies assessing asymptomatic populations reported lower prevalence rates ranging from 6 to 10%. Similarly, corpus-involving GA was found in up to 20% of symptomatic patients, compared to 6% to 8% in asymptomatic groups (Fig. 4b). These findings suggest that symptoms may be an important indicator of underlying atrophic changes. This also has implications for screening programs, which by definition will be assessing asymptomatic populations. These findings also stress the importance of lesion recognition for endoscopists performing gastroscopies, as GA appears to be very prevalent in patients reporting upper gastrointestinal symptoms. Early detection of GA in this cohort may be seen as a form of opportunistic screening.

The implications of these findings are substantial. The higher-than-expected prevalence of GA, particularly corpus involvement, raises important questions about current screening and surveillance strategies in these Northern and Western European countries. While historically GC screening programs in these populations were considered uneconomical due to the relatively low GC prevalence, these updated figures support the need to revisit such programs, which could target early detection of GA as a means to detect GC at an earlier and more treatable stage. Moreover, the data underline the importance of further research into the progression of distal GA to extensive (corpus-involving) GA, as well as the timeline of such progression [43].

This study has several limitations, most notably the significant heterogeneity observed across analyses. To explore potential sources, we conducted a meta-regression using the Meta-Essentials package, examining moderators including publication year, sample size, study design (retrospective vs. prospective), and assessment method (histological vs. serological) (Appendix 3).

Results showed that effect sizes declined slightly but significantly over time (β =  − 0.005, 95% CI [− 0.009, − 0.001], *P* = 0.020) and with larger sample sizes (*β* =  − 0.00001, 95% CI [− 0.00002, − 0.000001], *P* = 0.030), suggesting smaller, older studies may overestimate effects. Prospective designs were associated with lower (though non-significant) effect sizes compared to retrospective ones (*β* =  − 0.05, *P* = 0.090). Histological assessment yielded slightly higher effect sizes (*β* =  + 0.04, *P* = 0.048), while serological assessment was linked to slightly lower values (*β* =  − 0.03, *P* = 0.060).

The observed trends may reflect improved rigour and reduced publication bias in newer, larger studies. Future research should therefore aim to use larger sample sizes to minimize overestimation and increase statistical power, favor prospective designs to minimize bias, and employ histological assessments when feasible, as these tend to yield more valid effect size estimates. By adopting these approaches, the reliability and generalizability of findings in this field can be enhanced.

There was also considerable heterogeneity among the included studies in terms of histological classifications and serological biomarkers used. Pepsinogen I, a key biomarker for corpus atrophy, had widely varying cutoff values across studies, from < 17 to < 150 µg/L. While the inclusion of serological studies could be seen as a limitation of this study, it was crucial to highlight the contrast between diagnostic methods, especially as reliance on serological assessments continues to grow. Our analysis suggests that such reliance may lead to underreporting of the true prevalence of GA and corpus GA in particular. We were unable to control for significant risk factors, such as smoking, *H. pylori* status and family history, for both GA and GC across the studies, which again was evidenced by the high I^2^ values reported. As reported in Appendices 1 and 2, funnel plot analysis also suggested a risk of publication bias, which highlights the need for further well-designed studies, controlled for relevant risk factors, to be conducted.

This study is the first systematic review and meta-analysis to comprehensively assess the prevalence of GA in Western and Northern European populations. Unlike the study by Mulder et al. [11], we included studies that assessed prevalence using serological testing. This method is gaining popularity, and we considered it important to evaluate the differences in prevalence rates reported through serological versus histological assessment.

## Conclusion

This study demonstrates that the prevalence of GA, both overall and specifically involving the corpus, may be higher than previously reported in Northern and Western European populations. These findings challenge long-held assumptions about the epidemiology of GA in these regions and suggest that current estimates may underestimate the true burden of disease, particularly when serological methods are used. Given the potential increased prevalence, especially of the higher-risk corpus involvement, these results warrant renewed consideration of targeted screening and surveillance programs aimed at identifying GC precursors at an earlier, more treatable stage. Introducing such measures in selected populations could have important implications for improving long-term outcomes in GC through earlier detection and intervention.

## Data Availability

No datasets were generated or analysed during the current study.

## Appendix 1

Funnel Plot Analysis 

Funnel plot analysis of studies reporting the prevalence of GA regardless of topographical distribution in the stomach. Asymmetry was noted, suggesting publication bias. Adjusted combined effect size is closer to zero, which suggests the reported pooled effect may be overestimated due to missing negative or null results. Egger’s regression test indicated the intercept was significantly different from zero (intercept = 6.50, 95% CI: 2.19–10.82, *P* = 0.005), indicating the presence of small-study effects. This finding supports the visual asymmetry observed in the funnel plot.

## Appendix 2

Funnel plot analysis of studies reporting the prevalence of GA affecting the corpus of the stomach. Asymmetry was noted, suggesting publication bias. Adjusted combined effect size is closer to zero, which suggests the reported pooled effect may be overestimated due to missing negative or null results. Egger’s test for funnel plot asymmetry indicated a non-significant trend toward publication bias (intercept = 2.22, *P* = 0.081). While not statistically significant at the conventional level, this finding, combined with visual funnel plot asymmetry and trim-and-fill results, suggests possible small-study effects or publication bias.

## Appendix 3

**Table:**

Moderator	Type	Coding	β coefficient	95% CI	*P*-value	Interpretation
Publication year	Continuous	Actual year (e.g., 2000, 2021)	− 0.005	[− 0.009, − 0.001]	0.020	Effect sizes slightly decrease over time
Sample size	Continuous	Number of patients	− 0.00001	[− 0.00002, − 0.000001]	0.030	Larger studies report smaller effect sizes
Retrospective design	Categorical	0 = Retrospective, 1 = Prospective	− 0.05	[− 0.10, 0.01]	0.090	Prospective studies tend to report lower effects
Histological assessment	Categorical	0 = No, 1 = Yes	+ 0.04	[0.00, 0.08]	0.048	Studies using histology show higher effect sizes
Serological assessment	Categorical	0 = No, 1 = Yes	− 0.03	[− 0.06, 0.00]	0.060	Serology-only studies show slightly lower effects

Meta-regression results for listed moderators. Meta-regression analyses revealed several factors influencing reported effect sizes. Effect sizes showed a slight but significant decline over time (*β* =  − 0.005, 95% CI [− 0.009, − 0.001], *P* = 0.020), indicating that more recent studies tend to report smaller effects. Similarly, larger sample sizes were associated with reduced effect sizes (*β* =  − 0.00001, 95% CI [− 0.00002, − 0.000001], *P* = 0.030), suggesting that smaller studies may overestimate effects. Although not statistically significant, prospective study designs tended to report lower effect sizes compared to retrospective designs (*β* =  − 0.05, 95% CI [− 0.10, 0.01], *P* = 0.090). Studies employing histological assessment reported slightly higher effect sizes (*β* =  + 0.04, 95% CI [0.00, 0.08], *P* = 0.048), while serological assessment was associated with marginally lower effect sizes (*β* =  − 0.03, 95% CI [− 0.06, 0.00], *P* = 0.060).
